# A Decision Support System (DSS) for the Prediction and Selection of Optimum Operational Parameters in Pressure Die-Casting Processes

**DOI:** 10.3390/ma15155309

**Published:** 2022-08-02

**Authors:** Juan Martínez-Pastor, Juan José Hernández-Ortega, Rosendo Zamora

**Affiliations:** Departamento de Ingeniería Mecánica, Materiales y Fabricación, ETSII, Universidad Politécnica de Cartagena, E-30202 Cartagena, Spain; jm.pastor@upct.es (J.M.-P.); rosendo.zamora@upct.es (R.Z.)

**Keywords:** pressure die-casting, decision support system, process optimisation

## Abstract

A large number of material and process parameters affect both the part quality and the process performance in pressure die-casting (PDC) processes. The complex relations between most of these variables make PDC process optimisation a difficult issue which has been widely studied for many years. Although there are several analytical and numerical models to optimise certain process parameters, it is difficult to establish a specific operational configuration for PDC machines that ensures the joint optimisation of these variables. Therefore, in this study, some of these optimisation models have been implemented in a Decision Support System (DSS) that allows us to define an operational region that establishes a setup of machine parameters that ensures the manufacture of quality parts. By using this DSS, the user can set the values of the input variables related to the casting material, the die, or the casting machine. Then the corresponding calculations are made by the system and the results are expressed in terms of certain output variables such as the maximum filling time, maximum filling fraction, or the plunger velocity profile among others. The DSS allows the user to estimate the influence between input and output variables and find proper values for the input variables to achieve an optimum operational range. Consequently, improved process performance can be achieved taking into account productivity, part quality, and economic aspects.

## 1. Introduction

The pressure die-casting process is one of the most relevant manufacturing systems employed in engineering, since it can produce a wide range of products with a good surface finish, high quality, and dimensional accuracy [1]. Due to the widespread use of this casting technique, especially in powerful industrial sectors, such as automotive or aerospace [2], the optimisation of this manufacturing process acquires great importance in terms of part quality, productivity, and costs.

In pressure die-casting machines, many interconnected process and material parameters must be properly adjusted to optimise the process performance. Therefore, the quality of a casting is strongly related to certain variables: initial molten metal temperature, initial die temperature, filling fraction, first and second stage plunger velocities, accumulator pressure, and the design of the gating, cooling and venting systems [3]. Although this is a well-known manufacturing process using mature technology, certain challenging issues still remain unresolved, especially in terms of part quality. The most critical common casting defects are those related to gas entrapment (porosity), shrinkage, and lack of filling in the thinnest sections, which are responsible for the majority of casting defects [4,5,6,7].

For this reason, a better understanding of the interaction between all these process parameters and their effect on product quality and on process performance and costs is a current core field of research. In a first group of works [8,9,10,11,12,13], one can find experimental studies that analyse the influence of a limited set of process variables on casting quality. Dargusch et al. [8] implemented a series of pressure sensors in the cavity to measure the influence of the intensification pressure and casting velocity on the porosity level of the parts. Moreover, Laws et al. [9] developed a study where the process parameters of initial melt temperature, injection pressure, and injection velocity were analysed from the empirical data, finding an optimum operational window. In a similar way, several authors have also conducted quantitative measurements or used image analysis techniques to study the influence of injection pressure and melt temperature on casting porosity [10,11,12,13].

The optimisation of pressure die-casting processes has also been performed using analytical methods for computing certain process parameters. Two studies which should be mentioned are the works of Faura et al. [14] and López et al. [15], where a plunger acceleration law for the slow shot stage, based on the shallow-water approximation, was employed to establish the critical plunger velocity that provides the minimum air entrapment into the cavity by adjusting the filling level in the shot sleeve. As a result of the use of this plunger acceleration law, an operating range can be established which minimizes both the filling time and the porosity of the parts. Fiorese et al. [16] published a similar work, where the analytical treatment of the plunger motion profile was used to predict the best processing conditions for reducing porosity and consequently improving the tensile strength of the castings.

Another approach employed to optimise the pressure die-casting process is based on the implementation of Taguchi’s quality technique, since it allows the evaluation of the effect of process parameters on the quality properties of the castings. Anastasiou [17] studied the influence of initial melt temperature, initial die temperature, first and second stage plunger velocities, and third stage pressure in porosity formation. Similar studies and further work following that approach were developed adding other methodologies [18,19,20,21,22,23].

In addition, numerical simulation techniques have been applied to the analysis and optimisation of pressure die-casting processes. Mention should be made of the works by Khayat [24] and Barone and Caulk [25], where numerical models were applied for the prediction of air trapped in the parts. Similarly, the VOF (Volumen of Fluid) method was successfully used for the same purpose in the studies carried out by Zhao et al. [26] and Hernández-Ortega et al. [27]. In the work of Hu et al. [28], experimental and numerical data were employed to predict different designs of the runner and the gating system. In further studies, CFD (Computational Fluid Dynamics) was also used to analyse the influence of acceleration parameters and the filling fraction on air entrapment in the shot sleeve [29,30] to evaluate the critical velocity in the shot sleeve [31], to carry out the thermal analysis of the pressure die-casting process [32,33,34], or to evaluate the overall process performance with CAE (Computer Aided Engineering) [35,36].

Modern engineering tools based on artificial intelligence models, such as abductive networks and neural networks [37,38,39], are also being used in the optimisation of casting processes. Recently, other artificial intelligence techniques, such as genetic algorithms, in combination with certain optimisation tools have been shown to be efficient methods to perform real-time and very accurate predictions of the optimal processing conditions [40,41,42].

Finally, Decision Support Systems (DSS) are also interesting and effective optimisation tools that have been developed in recent years to assist decision making in manufacturing engineering, for either designing, planning, or operating production systems [43,44,45,46]. Those DSS based on hierarchy analysis techniques present several advantages to optimise the process performance, managing both technical and economic/costs decision models with a relational database for selecting the most efficient processing conditions in terms of savings in resources and time during the design and process selection tasks [47,48,49].

Except for the work of Karni [50], most models described in the literature aim to maximise part quality (decreasing the quantity of trapped air or the porosity, avoiding premature solidification among others), independently, by optimising or focusing on certain process parameters, such as plunger velocity of the slow shot stage, melt temperature, injection pressure, or fill fraction; but the selection of the whole parameters in the die-casting process and the limitations of the machine are not taken into account. Therefore, the creation of a specific procedure on how to obtain compromise solutions for all operational parameters is not addressed enough due to the high number of variables involved.

Previous experience of our research group in DSS development [47], and the work carried out on the definition of analytical, numerical, and experimental models in die-casting processes on the plunger movement in the slow shot phase [12,14,15,29], as well as the numerical and experimental study of die cavity filling [27], has led us to the development of a DSS capable of addressing this problem.

The present work reports the development and implementation of a DSS for the selection of the optimum operational window and the prediction of process performance for pressure die-casting processes, taking into account the capabilities of the machine. Herein, the parameters related to the molten metal, the shot sleeve (filling fraction, plunger acceleration profile, and injection pressure), the thermal properties of the die and the geometry of the gating system have been implemented.

## 2. Methodology for the Selection of Operational Parameters in the Pressure Die-Casting Machines

**Figure 1:**
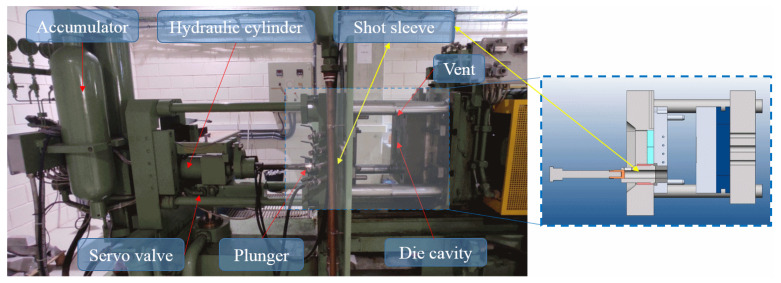
Photography of the main mechanical devices in pressure die-casting machines.

Pressure die-casting machines with a cold chamber, are usually employed for the manufacture of high-quality parts with complex shapes made of copper, zinc, magnesium, and aluminium alloys [3]. They operate under the action of a hydraulic cylinder, which pushes and injects the molten metal, which is previously poured into the shot sleeve, towards the die cavity at pressures of up to 70 MPa (Figure 1).

In this current study, the previous work carried out by our research group on the plunger acceleration law and the minimum filling fraction that minimizes air entrapment [12,14,15,29] has been integrated into this DSS together with certain models related with injection and distribution systems [49,50], gas evacuation system [51], and adjustment of operational limits [52,53,54].

**Figure 2:**
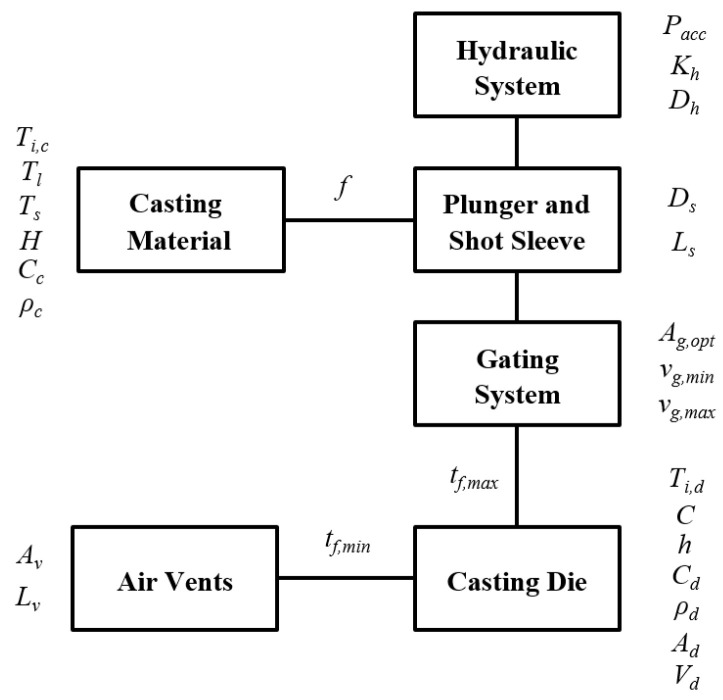
Block diagram of the systems and process variables in pressure die-casting machines.

Figure 2 shows the block diagram that includes the main process and material parameters related to such models.

The following inputs and outputs have been taken into account within the DSS and its associated optimisation method:

- Inputs: accumulator pressure ($P_{acc}$); hydraulic loss constant ($K_{h}$); hydraulic cylinder diameter ($D_{h}$); plunger/shot sleeve diameter ($D_{s}$); shot sleeve length ($L_{s}$); casting temperature ($T_{i,c}$); liquidus and solidus temperatures ($T_{l}$, $T_{s}$); heat of fusion (*H*); casting heat capacity ($C_{c}$); casting density ($\rho_{c}$); minimum gate velocity ($v_{g,min}$); maximum gate velocity ($v_{g,max}$); initial die temperature ($T_{i,d}$); die constant (*C*); heat transfer coefficient (*h*); die heat capacity ($C_{d}$); die density ($\rho_{d}$); cavity surface area ($A_{d}$); cavity volume ($V_{d}$); cross-section area of the vent ($A_{v}$); vent length ($L_{v}$).
- Outputs: plunger velocity profile (X′(t)); minimum plunger/shot sleeve diameter ($D_{s,min}$); optimum cross-section area of the gate ($A_{g,opt}$); minimum cross-section area of the vent ($A_{v,min}$); filling fraction (*f*); maximum filling fraction ($f_{max}$); minimum filling time ($t_{f,min}$); maximum filling time ($t_{f,max}$).

Two kinds of variables can be distinguished from among these parameters: soft variables are those that can be modified by an actuator or by the control unit (e.g., $P_{acc}$, $T_{i,d}$, $T_{i,c}$), whilst hard variables are those that require modifications in the preparation of the machine or the casting die ($D_{s}$, $A_{g}$, location and geometry of vents, etc.).

In this way, the objective of this methodology is to use this DSS to define, in a few iterations, an adequate combination of soft variables that maximise flexibility, together with the hard variables that guarantee the adequate performance of the PDC machine to manufacture a certain piece of aluminium. The models implemented in the DSS are detailed in the following sections.

### 2.1. Optimisation Models Implemented in the DSS

#### 2.1.1. Process Performance: $PQ^{2}$

The $PQ^{2}$ model was established in the works developed by Karni [50] and Pego [52] and can be used to obtain those parameters related to the gate system and the plunger. The $PQ^{2}$ analysis of fluid flow in die-casting is based on an energy balance. This model can describe the heat transfer that occurs between the molten metal and the cavity surfaces, starting from the premises of unidirectional flow, uniform metal and die temperature, and linear release of latent heat during metal solidification. The main advantage of this approach is that it enables the assessment of how the variation of these parameters affects the process performance.

**Figure 3:**
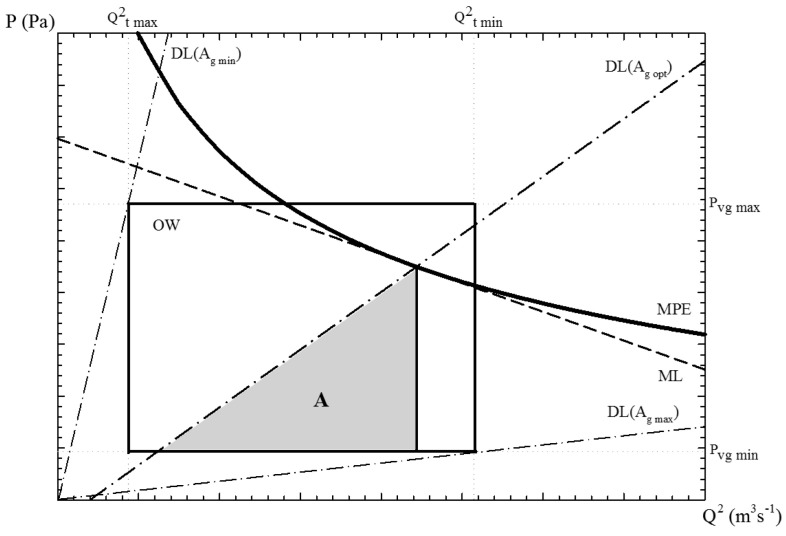
Conceptual $PQ^{2}$ diagram, including the representation of OW, DL, MPE, and ML. The area (*A*) of the triangle is a measure of the flexibility.

Therefore, in a $PQ^{2}$ diagram (Figure 3), the “Machine Line” (ML) is associated with the capacity of the hydraulic system to apply pressure from the accumulator ($P_{acc}$), the “Die Line” (DL) represents the casting die resistance for a given cross-section area of the gate ($A_{g}$), and the “Machine Performance Envelope” (MPE) expresses the maximum energy that can be applied during the process. The operational window (OW) is given by the minimum ($t_{f,min}$) and maximum ($t_{f,max}$) filling times, together with the minimum ($v_{g,min}$) and maximum gate velocities ($v_{g,max}$).

The flexibility of the pressure die-casting process is defined as the experimental variation capacity of the soft variables within OW and below MPE, measured by the area *A*, and constitutes the range of optimum values of filling time and gate velocity.

#### 2.1.2. Product Quality: Determination of the Optimum Shot-Sleeve Velocity Profile

**Figure 4:**
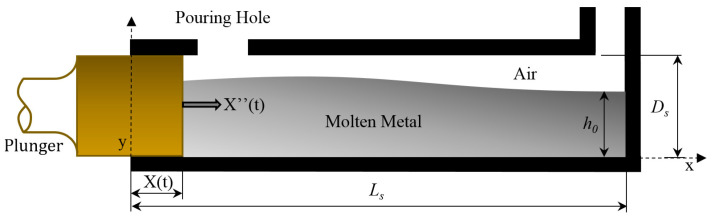
Schematic representation of plunger and shot sleeve of a pressure die-casting machine and the parameters related to the plunger acceleration law.

As previously mentioned, an optimised plunger acceleration law prevents the appearance of the turbulent flow causing gas entrapment during the injection phase. The studies carried out by Faura et al. [14] and López et al. [15] established a critical plunger velocity that minimizes the air entrapment during the shot phase (Figure 4). For plunger velocity values that are higher than the critical value, the wave of molten metal would touch the ceiling of the shot sleeve before beginning to break. For values that are lower than the critical value, the same effect is produced at the rear side of the shot sleeve [29].

The advantage of using this plunger acceleration law is that it enables the amount of trapped air in the shot sleeve to be predicted for a given set of processing conditions, which can consequently be optimised. For this reason, this model of plunger acceleration law has been employed to minimize the amount of air trapped in the cold chamber from a specified filling fraction (*f*) and geometry of the shot sleeve with its analytical expression being [14]:

(1) $$X^{{}^{\prime \prime }}\left(t\right)=\frac{2\cdot \alpha^{2}}{3\cdot \beta }\cdot {\left(1-\frac{\alpha \cdot t}{\beta }\right)}^{-4/3}$$

where α is defined as $\sqrt{g\cdot h_{0}}$ and β is a positive constant to be fitted.

#### 2.1.3. Operational Costs: Determination of the Filling Fraction

To carry out a pressure die-casting process that produces high quality parts with a good surface finish, an optimised pouring volume (or filling fraction) is required to prevent part defects related to porosity without involving an excessive economic impact [27]. When estimating the minimum filling fraction, it should be taken into account that the higher the filling fraction, the lower the plunger acceleration, resulting in a longer processing time and thus a higher operational cost.

In this method, a criterion to establish the maximum filling fraction has also been imposed; the wave of molten metal must not reach the ceiling of the shot sleeve before the plunger surpasses the pouring hole. Assuming this condition, the maximum filling fraction can be determined by numerical resolution of the aforementioned plunger acceleration law. Taking the variable $X_{p}$ as the initial distance between the plunger and the pouring hole, the following relation can be applied [14]:

(2) $$\frac{X_{p}}{L_{s}}=1-3\cdot f_{max}+2\cdot f_{max}^{3/2}$$

Finally, the estimation of the operating time, which is significantly related to the production rate and energy costs, has also been taken into account. The estimations of the minimum filling time ($t_{f,min}$) and the maximum filling time ($t_{f,max}$) presented in the following section have been used to optimise the overall operating time [14,50].

#### 2.1.4. Operational Window: Fitting of the Operating Limits

To fully address the optimisation of PDC processes, it is also necessary to fit the operating limits of the OW introduced by the gating and vent systems. These process constraints are the minimum gate velocity ($v_{g,min}$), the maximum gate velocity ($v_{g,max}$), the minimum time required to evacuate the air inside the die cavity ($t_{f,min}$), and the solidification time of the casting material ($t_{f,max}$).

The minimum gate velocity ($v_{g,min}$) sets the limit above which porosity formation in the part is prevented, as well as the possibility of premature solidifications in the gating system. To set the value of this parameter, the condition of obtaining an atomized flow at the die entrance gate is imposed depending on the casting density and the gate thickness, according to the estimations recommended by NADCA (North American Die Casting Association, IL, USA) [53,54].

The maximum gate velocity ($v_{g,max}$) is related to the thickness of the gating system, which limits the maximum flow rate to feed the cavity. The value of this constraint is also estimated according to the values recommended by NADCA [54].

The minimum filling time is defined as that which allows the wind to evacuate the gas from the cavity without reaching sonic block conditions in the outlet section of the vent ($M_{v,out}\leq 1$), avoiding the appearance of shock waves. To estimate this parameter, the Bar-Meir model [51] has been used, which considers that the airflow inside the vent develops in quasi-stationary and isentropic conditions, whilst heat transfer effects are negligible. Therefore, the minimum cross-section area of the vent ($A_{v,min}$) can be determined by imposing $M_{v,out}=1$, which provides the minimum filling time ($t_{f,min}$).

The solidification time of a casting determines the appearance of premature solidifications in the gating system and the cavity, considering that the heat flow is unidirectional and the heat loss is linear [55]. Hence, the solidification time is used as a constraint to set the maximum filling time ($t_{f,max}$) considered in the PDC process.

### 2.2. Procedure for Selection of Operational Parameters: Maximisation of Process Flexibility

When the parameters of OW, ML, DL, and MPE are varied, the flexibility, which is used by the DSS to search for the optimal operational conditions, is modified. The proposed optimisation method only employs the DSS to modify the input variables of the $PQ^{2}$ model to find better processing conditions. After a few iterations, the values of the output variables are those that optimise process performance, product quality, and operational costs.

Therefore, the objective of this optimisation method implemented in the DSS is: first, the determination of the hard variables that generate the largest OW, and second, to maximise the flexibility *A* (the formulation of *A* can be found in [50]), thus making it easier to adjust the soft variables within this region to set the optimum operating point.

Consequently, the optimisation method consists of finding the OW and the slope of DL that maximises *A*, so that the DL crosses the lower left-hand corner and the upper right-hand corner of the OW. The optimum processing conditions are achieved when the plunger diameter ($D_{s}$) is the minimum required by the system, and the accumulator pressure ($P_{acc}$) provides the largest flexibility.

**Figure 5:**
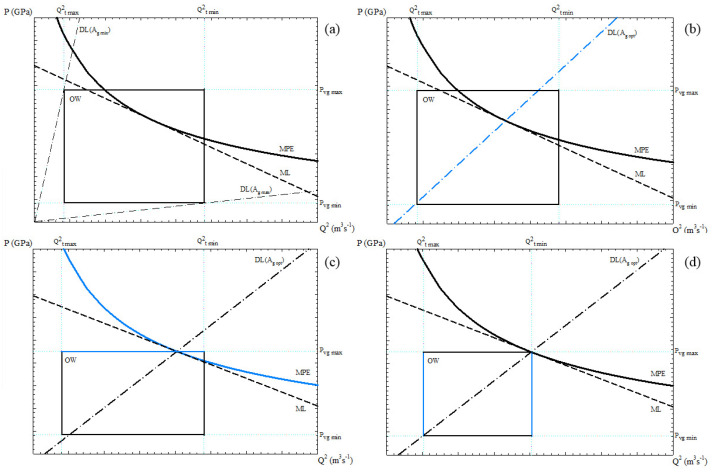
Qualitative representation of the optimisation procedure to be performed using the DSS: (**a**) step 1; (**b**) step 2; (**c**) step 3; (**d**) steps 4 and 5.

The procedure for selecting operational parameters implemented in the DSS consists of the following steps, which have been represented qualitatively and graphically in Figure 5:

(1) The input data are entered into the DSS, and the initial value of $D_{s,min}$ and $f_{max}$ are obtained (Figure 5a).
(2) The available $D_{s}^{*}$ closest to $D_{s,min}$ that matches the maximum filling fraction constraint is selected, and new values of $A_{g,opt}$, $A_{v,min}$, $f_{max}$ and $X^{\prime }(t)$ are recalculated (Figure 5b).
(3) Increasing the accumulator pressure ($P_{acc}$) below the maximum allowable and fitting the maximum gate velocity ($v_{g,max}$) to the intersection point between DL($A_{g,opt}$) and ML (Figure 5c).
(4) Increasing the minimum filling time ($t_{f,min}$) so that DL($A_{g,opt}$) cuts to OW in the upper right-hand corner (Figure 5d).
(5) Reducing the maximum filling time ($t_{f,max}$) or increasing the minimum gate velocity ($v_{g,min}$) so that DL($A_{g,opt}$) cuts to OW in the lower left-hand corner (Figure 5d).

As a result, such OW limits ensure the minimization of porosity and the absence of premature solidification in the casting die, while defining a region of maximised flexibility that allows the selection of the appropriate operational parameters for the PDC machine. An example of the practical application of this procedure is shown later in Section 4.

## 3. Management of Inputs and Analysis of Outputs with the DSS

Microsoft Visual Studio IDE was used for the development of this application due to its flexibility to modify and edit any variable or function. The DSS presented in this work has been conceived to predict the optimal values of process parameters in a short time and in a simple way.

The aim of using this DSS is to determine the optimal value of process parameters by evaluating the generated results. Therefore, the initial choice of process parameters (inputs) can be modified in each iteration of the method to analyse the change in the results (outputs). In this way, the fundamental aspects of the process (process performance, part quality, and operational cost) can be evaluated until the optimal process conditions are reached.

### 3.1. DSS Workflow

**Figure 6:**
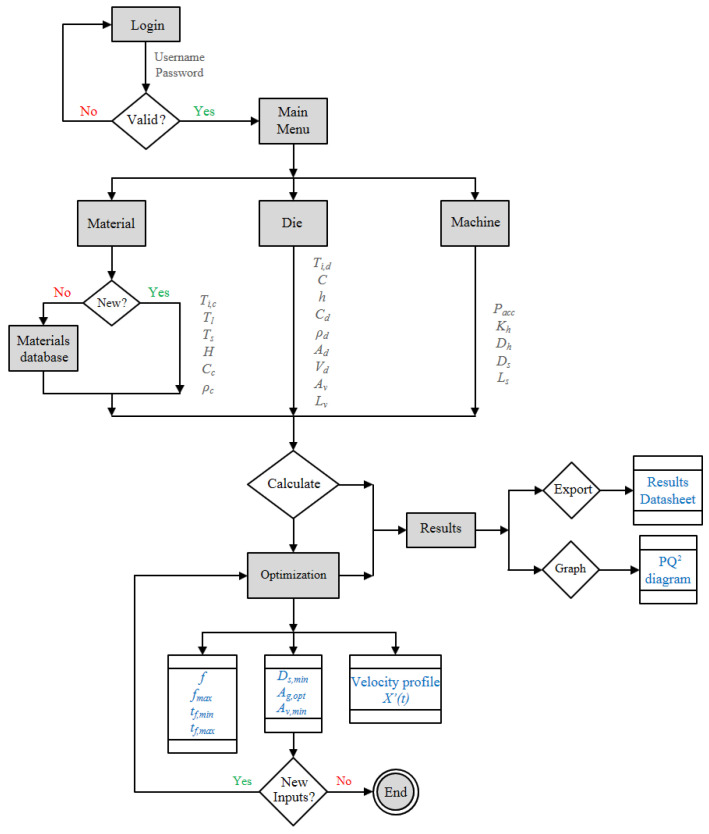
Flow diagram for the use of the DSS, including menus (blocks), input (in grey), and output (in blue) variables, and the workflow (connectors).

Figure 6 shows a flow diagram with the breakdown of the workflow associated with the developed DSS application. This application consists of three modules:

(1) DB (Data Base): contains all the information of the casting process.
(2) CW (Calculation Window): performs the calculations to obtain first ML, then DL, and finally MPE, from which the optimal values of the output variables (X′(t), $D_{s,min}$, $A_{g,opt}$, $A_{v,min}$, *f*, $f_{max}$, $t_{f,min}$, and $t_{f,max}$) are extracted.
(3) OM (Optimisation Module): takes the optimisation equations to provide optimised values of the input variables ($D_{s}$ and $P_{acc}$).

Once the application is started, the first menu that appears is the “Login” window, where the user has to enter a valid username and password. After the input of credentials, the main window of the program is shown, where the four menus of the application appear.

Process data are then entered for the first time in the “Casting Material” menu. The following material parameters must be specified as input variables; initial casting temperature ($T_{i,c}$), liquidus temperature ($T_{l}$), solidus temperature ($T_{s}$), heat of fusion (*H*), heat capacity ($C_{c}$), and casting density ($\rho_{c}$). These parameters can be uploaded automatically selecting a casting material previously saved in the program database, or manually if the casting material is not contained in it.

The next module of the main menu is “Casting Die”, where the parameters corresponding to the properties and geometry of the cavity and the vent must be specified. These variables are the following; initial die temperature ($T_{i,d}$), die constant (*C*), heat transfer coefficient (*h*), die heat capacity ($C_{d}$), die density ($\rho_{d}$), cavity surface area ($A_{d}$), cavity volume ($V_{d}$), vent cross-section area ($A_{v}$), and vent length ($L_{v}$).

The last module of this DSS is dedicated to process optimisation, which has five inputs to be defined; accumulator pressure ($P_{acc}$), hydraulic loss constant ($K_{h}$), hydraulic cylinder diameter ($D_{h}$), plunger diameter ($D_{s}$), and length of the shot sleeve ($L_{s}$). In this module, the value of the plunger diameter ($D_{s}^{*}$) can be selected among those available for the casting machine.

Once parameters have been correctly specified in these three menus of the application, the user should click on “Calculate”, and then the “Results” window appears, providing the initial values of the outputs:X′(t), $D_{s,min}$, $A_{g,opt}$, $A_{v,min}$, *f*, $f_{max}$, $t_{f,min}$, and $t_{f,max}$. At this point, step 1 of the optimisation procedure is completed.

### 3.2. Analysis of Results and Process Optimisation

These predicted results may not be definitive because these parameters can be edited to find the optimal solution of the PDC system. Once the initial calculations have been performed by the DSS, the “Graph” option is available and generates an additional window where the resulting $PQ^{2}$ diagram is displayed. Selecting the “Export” button, the application saves and provides a datasheet with the numerical results.

At this point, the “Optimisation” menu is available, and the user can carry out successive adjustments of the PDC system by specifying new values of available plunger diameter ($D_{s}^{*}$) and accumulator pressure ($P_{acc}$) but also of the OW limits ($t_{f,min}$, $t_{f,max}$, $v_{g,min}$, and $v_{g,max}$).

Thus, the available plunger diameter $D_{s}^{*}$ immediately greater than the minimum plunger diameter $D_{s,min}$ calculated by the DSS provides the maximum flexibility *A*, whilst the increase of $P_{acc}$ for a given value of $D_{s}^{*}$ provides larger flexibilities but also higher values of $v_{g,min}$ and $v_{g,max}$. Hence, the setting of $P_{acc}$ is limited by such constraints, which must be carefully selected as described in Section 2.1.4 prior to the optimisation process.

Therefore, steps 2 to 5 of the optimisation procedure can be performed as described in Section 2.2 to obtain the maximised process flexibility *A* together with the optimal values of soft and hard variables, i.e., the optimum operating point of the PDC process under study.

Once the optimisation has been finished, reaching the optimal process conditions, the user can access the “Results” window, where the resulting $PQ^{2}$ diagram can be viewed and extracted using the “Graph” option. Finally, the plunger velocity profile data and a report with the resulting optimal values of the input and output variables can be obtained by clicking on the “Export” button.

## 4. Case Study Employing the DSS

**Figure 7:**
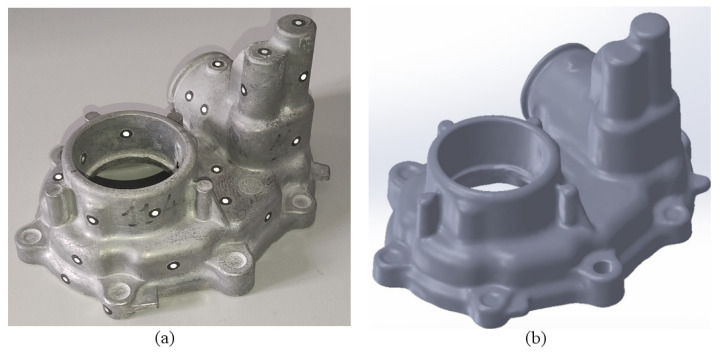
Photograph (**a**) and CAD file (**b**) of the motor housing employed for the case study.

In this section, a motor housing (Figure 7) made of the aluminium alloy A.413.0-F (Table 1) has been used as a case study to check this optimisation method through the developed DSS. Figure 7 shows a photograph and the CAD file of the part used. Table 2 includes the data corresponding to the case study that have been introduced into the DSS application menus of “Casting Material”, “Casting Die”, “Casting Machine”, and “Optimisation” described in the previous section.

**Figure 8:**
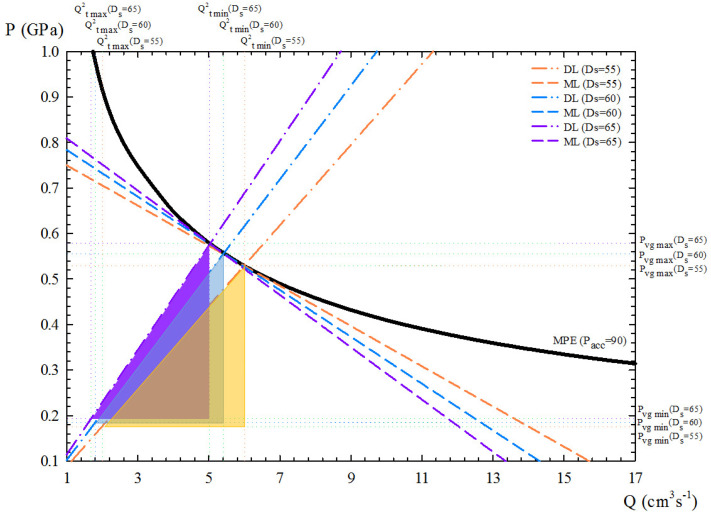
PQ${}^{2}$ diagrams obtained with the DSS for the case study for a given $P_{acc}$ = 90 bar, considering three different plunger diameters ($D_{s}$): 55, 60, and 65 mm. The areas of the triangles (flexibility) have been represented with different colours.

First of all, the influence of the plunger diameter $D_{s}$ is analysed. Plunger diameters of 55, 60, and 65 mm were checked, with 60 mm being the optimal result calculated by the DSS, which provides the largest flexibility for a given initial value of accumulator pressure (90 bar). These results are presented in Figure 8 in terms of the $PQ^{2}$ diagram. From these data, it can be deduced that below the maximum filling fraction, a higher filling fraction is achieved when taking the lowest available plunger diameter above the minimum value calculated by the DSS, which leads to a more productive process.

**Figure 9:**
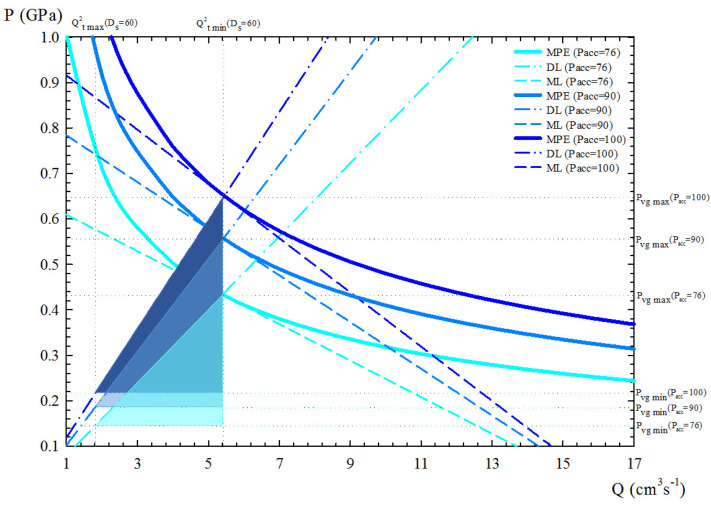
PQ${}^{2}$ diagrams obtained with the DSS for the case study for a given $D_{s}$ = 60 mm, considering three different accumulator pressures $\left(P_{acc}\right)$: 76, 90, and 100 bar. The areas of the triangles (flexibility) have been represented with different colours.

Second, the accumulator pressure $P_{acc}$ was also considered to study process performance. Three different values were taken into account: 76, 90, and 100 bar. The corresponding results are presented in Figure 9, again in terms of the $PQ^{2}$ diagram. These data show that for a selected plunger diameter (60 mm), reducing the accumulator pressure (76 bar) leads to lower energy consumption and shorter flexibility, while increasing it (100 bar) leads to higher energy consumption and greater flexibility but also higher values of minimum and maximum gate velocities.

Regarding the flexibility of the PDC machine for this aluminium part in terms of the PQ${}^{2}$ diagram units, the DSS provides flexibility values between 510 and 764 (Figure 8 and Figure 9). This represents differences of 33.25% in terms of flexibility for different choices of $P_{acc}$ and $D_{s}$, with a maximum value corresponding to the combination of $P_{acc}$ = 100 and $D_{s}$ = 60 mm.

Finally, for this case study, taking the maximisation of flexibility against energy consumption as the optimisation criterion, a plunger diameter of 60 mm and an accumulator pressure of 100 bar have been selected as optimal input parameters. For these process parameters, the values of minimum and maximum gate velocities were fitted to 1.74 ms${}^{-1}$ and 3 ms${}^{-1}$, respectively, during steps 4 and 5 of the optimisation procedure. For this optimisation, an available vent with a cross-section area of 18 mm${}^{2}$ can be selected in accordance with the calculated value of $A_{v,min}$. For the operating conditions considered, the porosity levels may increase by up to 70% if the maximum plunger speed is not appropriately selected [12].

Therefore, for this case study, the maximisation of flexibility gives us the optimal input parameters $D_{s}$ = 60 mm and $P_{acc}$ = 100 bar, for which the DSS provides the specific values of the available auxiliary elements of this PDC machine (hard variables) so that the appropriate and reliable combination of gate section ($A_{g}$), vent section ($A_{v}$), and filling fraction (*f*) is achieved. This machine setup, together with the adjustment of $X^{\prime }\left(t\right)$ as the main soft variable, ensures that these aluminium parts are manufactured with very low porosity, without a premature solidification, and with a reduced operating cost.

The results given in Table 3 show the corresponding optimal values of the outputs, while Table 4 shows the resultant optimum plunger velocity profile $X^{\prime }(t)$.

## 5. Conclusions

This paper has presented a tool for the selection of operational parameters that ensures quality castings in pressure die-casting machines. This allows the user to choose the best operational setup to manufacture a certain part on a given PDC machine. To optimise process parameters, this DSS first employs the $PQ^{2}$ model to define an initial operating window. Second, the plunger acceleration law is used to search for an optimal operating window which ensures the minimization of the air entrapment and to determine the maximum filling fraction that limits the operating time, reducing operating costs.

The advantages of using the DSS developed in this study are the prediction of optimised process parameters and the testing of different operating conditions. The main novelty of this DSS is that it takes into account the multiple available configurations of a PDC machine during the decision process.

In addition, when considering diverse operational conditions, the influence of the material or process variables can be analysed and quantified in terms of process performance and product quality. With regard to the handling of the DSS, it also includes a help icon which is available in each menu of the software and which provides a dialog box with the necessary information. The results of the DSS can be converted into the usual text file formats, be viewed in the application, or be exported to a folder.

This DSS is currently being improved by incorporating new optimisation routines to automate the current iterative process. We are also considering the inclusion of a simulation module that can provide optimal geometrical solutions in terms of the layout of the gates and the vents.

## Figures and Tables

**Table 1:** Composition of the aluminium casting alloy (A.413.0-F/UNS A14130/EN 1706 AC 44100) considered for the case study.

Si (%)	Fe (%)	Cu (%)	Mg (%)	Mn (%)	Ni (%)	Zn (%)	Sn (%)
11.0–13.00	2.0	1.0	0.1	0.35	0.5	0.5	0.15

**Table 2:** Inputs employed for the case study in the DSS.

Casting Material		Casting Die		Casting Machine		Optimisation	
$T_{i,c}$ (${}^{\circ }$C)	680	$T_{i,d}$ (${}^{\circ }$C)	260	$P_{acc}$ (bar)	90	$P_{acc}$ (bar)	76, 90, 100
$T_{l}$ (${}^{\circ }$C)	582	*C*	71,593,693	$K_{h}$	1,000,000	$D_{s}$ (mm)	55, 60, 65
$T_{s}$ (${}^{\circ }$C)	574	*h* (W/m${}^{2}$K)	4000	$D_{h}$ (mm)	125	$v_{g,min}$ (m/s)	1.4
$C_{c}$ (kJ/kg${}^{\circ }$C)	0.963	$C_{d}$ (kJ/kg${}^{\circ }$C)	0.615	$D_{s}^{*}$ (mm)	45, 50, 55, 60, 65, 70	$v_{g,max}$ (m/s)	3.2
$\rho_{c}$ (kg/m${}^{3}$)	2660	$\rho_{d}$ (kg/m${}^{3}$)	7800	$L_{s}$ (mm)	273		
*H* (kJ/kg)	389	$A_{d}$ (mm${}^{2}$)	15490.5				
		$V_{d}$ (mm${}^{3}$)	256,500				
		$A_{v}$ (mm${}^{2}$)	18				
		$L_{v}$ (mm)	152				

**Table 3:** Results obtained by the DSS for $D_{s}^{*}$ = 60 mm, $P_{acc}$ = 100 bar, $v_{g,min}$ = 1.74 ms${}^{-1}$, $v_{g,max}$ = 3 ms${}^{-1}$.

Soft Variables		Hard Variables	
$P_{acc}$ (bar)	100	$D_{s}$ (mm)	60
$t_{f,min}$ (s)	0.112	$A_{g,opt}$ (cm${}^{2}$)	7.75
$t_{f,max}$ (s)	0.193	$A_{v,min}$ (mm${}^{2}$)	17.44
$X^{\prime }\left(t\right)$	Table 4	*f* (%)	39.88
		$f_{max}$ (%)	41.02

**Table 4:** Plunger velocity profile $X^{\prime }(t)$ obtained by the DSS for the optimal values of the outputs.

*t* (s)	*X*(*t*) (m)	*X*${}^{\prime }$(*t*) (ms${}^{-1}$)
0.000	0.000	0.0000
0.056	0.001	0.0017
0.112	0.004	0.0036
0.168	0.009	0.0056
0.224	0.018	0.0800
0.280	0.030	0.1070
0.336	0.046	0.1380
0.392	0.069	0.1760
0.448	0.101	0.2260
0.504	0.149	0.2960
0.560	0.257	0.4590

## Nomenclature

The following abbreviations are used in this manuscript:

α	First parameter of the plunger acceleration law
*A*	Measure of the flexibility
$A_{d}$	Surface area of the die cavity
$A_{g}$	Cross-section area of the gate
$A_{v}$	Cross-section area of the vent
β	Second parameter of the plunger acceleration law
*C*	Casting die constant
$C_{c}$	Heat capacity of the casting material
$C_{d}$	Heat capacity of the casting die
$D_{S}$	Plunger/Shot sleeve diameter
*f*	Fill fraction of casting material in the shot sleeve
$h_{0}$	Initial height of the molten metal in the shot sleeve
*h*	Heat transfer coefficient at the die cavity surface
*H*	heat of fusion
$K_{h}$	Characteristic constant of the hydraulic system
$L_{s}$	Length of the shot sleeve
$L_{v}$	Length of the vent
$P_{acc}$	Pressure exerted by the accumulator to the hydraulic system
*Q*	Flow rate
$\rho_{c}$	Casting material density
$\rho_{d}$	Die material density
$t_{f}$	Filling time of the casting material in the die cavity
$T_{i,c}$	Initial casting material temperature
$T_{i,d}$	Initial die temperature
$T_{l}$	Liquidus temperature of the casting material
$T_{S}$	Solidus temperature of the casting material
$v_{g}$	Gate velocity of casting material
$V_{d}$	Volume of the die cavity
$X_{p}$	Distance between plunger and pouring hole
X(t)	Position of the plunger in shot sleeve
$X^{\prime }\left(t\right)$	Plunger velocity profile
$X^{\prime \prime }\left(t\right)$	Plunger acceleration profile
CAE	Computer Aided Engineering
CFD	Computational Fluid Dynamics
DL	Die Line
DSS	Decision Support System
ML	Machine Line
MPE	Machine Performance Envelope
NADCA	North American Die Casting Association
OW	Operational Window
PCD	Pressure Die Casting
VOF	Volume Of Fluid
